# Relationship between ancient bridges and population dynamics in the lower Yangtze River Basin, China

**DOI:** 10.1371/journal.pone.0182560

**Published:** 2017-08-09

**Authors:** Yang Zhao, Xin Jia, Harry F. Lee, Hongqiang Zhao, Shuliang Cai, Xianjin Huang

**Affiliations:** 1 School of Geographic and Oceanographic Sciences, Nanjing University, Nanjing, Jiangsu Province, China; 2 Needham Research Institute, Cambridge, United Kingdom; 3 Department of Geography, The University of Hong Kong, Hong Kong, China; 4 Department of History, Xiamen University, Xiamen, Fujian Province, China; 5 Yixing Municipal Administration Commission Office of Cultural Heritage, Yixing, Jiangsu Province, China; Seoul National University College of Medicine, REPUBLIC OF KOREA

## Abstract

It has been suggested that population growth dynamics may be revealed by the geographic distribution and the physical structure of ancient bridges. Yet, this relationship has not been empirically verified. In this study, we applied the archaeological records for ancient bridges to reveal the population growth dynamics in the lower Yangtze River Basin in late imperial China. We investigated 89 ancient bridges in Yixing that were built during the Ming and Qing dynasties (AD1368–1911). Global Position System information and structure (length, width, and span) of those bridges was measured during our field investigations. Their distribution density was calculated by ArcGIS. The historical socio-economic dynamics of Yixing was inferred from the distribution and structure of ancient bridges. Based on the above information, the population growth dynamics in Yixing was projected. Our results show that 77 bridges were built in Yixing during the Qing dynasty, which is 6.41 times more than the number built during the Ming dynasty. In the Ming dynasty, bridges were built on pivotal routes; in the Qing dynasty, bridges were scattered across various places. Over the period, the density distribution of bridges shifted northwestward, while the average length and width of bridges decreased. The increasing number of bridges corresponded to population growth, largely attributable to massive clan migration from northern China during the Little Ice Age. The shift in the density distribution of bridges corresponded to the formation of settlements of large clans and the blossoming of Yixing Teapot handicrafts. The scattering and the reduction in average length and width of bridges was due to the dispersal of population and the associated formation of small settlements in the latter period. Our approach is innovative and robust, and could be employed to recover long-term historical population growth dynamics in other parts of China.

## Introduction

Ancient people settled along rivers or around lakes because of their dependence on water, and many important ancient civilizations are located along rivers, including the River Nile, River Ganges, Yellow River, and Yangtze River. Subject to population growth over time, water transportation was needed to facilitate the expansion of human settlements. The oldest known dugout canoe was invented about 10 ka years ago in Lincolnshire in England [1]. Since that time, humans sought a more convenient means to cross rivers and lakes. Consequently, bridges were built in plains areas where rivers and lakes crisscross, as those areas were ideal for human settlements. In turn, the building of bridges also helped to increase population density in the nearby area.

Population growth was considered as the underlying factor for social development and the outbreak of war [2]. The effects of population growth could be spread to society, the economy, the ecology, and the environment. Furthermore, population has been accepted as a major proxy in examining the triangulation among humans, society, and the environment in pre-industrial societies [3–5]. Population density can be estimated by statistical data, remote-sensing imagery, and historical documents [6–8]. With the advancement of new population estimation methods, we can develop more accurate estimates of population density in small regions (e.g., county or town). Nevertheless, subject to the deficiency of fine-grained historical demographic records, scholars must rely on archaeological remains to trace historical population growth dynamics [9–12].

With the increase of the global population from 360 million to 1,563 million in AD1300–1900, humans gradually occupied the entire world [13]. Population in China increased from 70 million to 432 million in AD1293–1851 [14–15]. Owing to the cold climate during the Little Ice Age (LIA, AD1450–1850), a large number of people migrated southward from colder northern China to the warmer Yangtze River Basin. The population in Jiangsu and Zhejiang (Jiangnan region) has exceeded the population in northern China since the Yuan dynasty (AD1271–1368) [16–18]. Such a drastic population expansion in the lower Yangtze River Basin during the LIA brought the enhancement of communication and transportation. Hence, increasing numbers of roads were built in Jiangnan, especially in the late imperial era [19]. When roads were blocked by rivers, bridges were built to help people cross. Generally, the construction of bridges was driven by the increasing level of communication and transportation in the regions adjacent to the rivers, which was dependent upon the population size.

Given the evidence cited above, it can be further supposed that historical population growth dynamics could be revealed by the geographic distribution and the structure (e.g., length, width, etc.) of ancient bridges [20]. However, this proposed relationship has never been verified empirically. In this study, we sought to recover the population growth dynamics on the basis of archaeological remains of ancient bridges (in terms of their geographic distribution and structural characteristics) in the lower Yangtze River Basin in late imperial China. We investigated 89 ancient bridges in Yixing, Jiangsu that were built during the Ming and Qing dynasties (AD1368–1911). The Global Position System (GPS) information and structure (length, width, and span) of those bridges were measured during our field investigations. The distribution density of these bridges was calculated by ArcGIS. The socio-economic dynamics of the Ming and Qing dynasties were inferred from the distribution and structure of ancient bridges. Finally, the population growth dynamics in Yixing was projected.

## Study area

**Fig 1:**
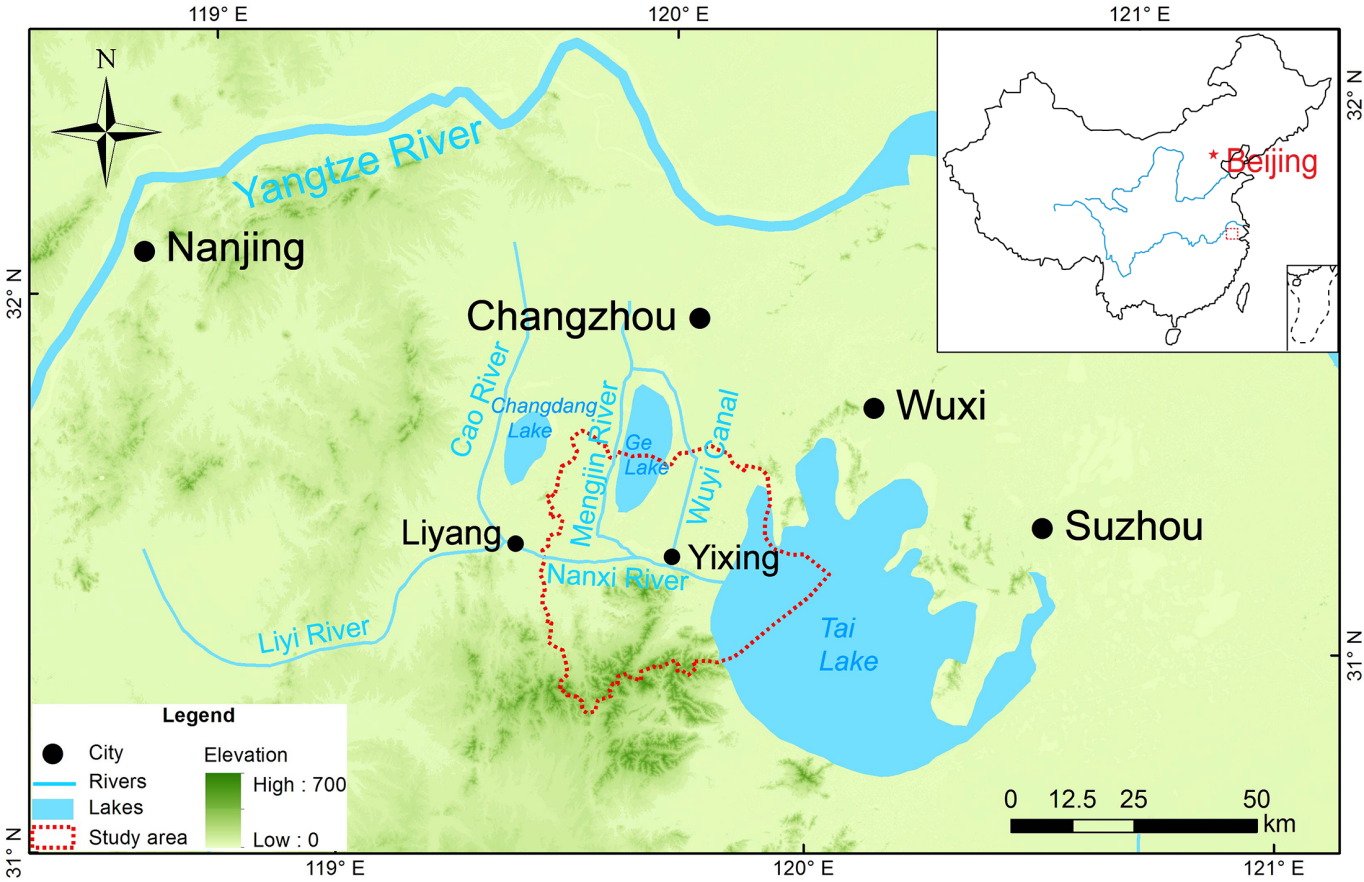
Location map of Yixing. The map was generated in ArcGIS version 10.1 (www.esri.com/software/arcgis). The topographic information was obtained from the Digital Elevation Model (DEM) with a solution of 90m× 90 m, which was downloaded from the Consortium for Spatial Information (CGIAR-CSI) (http://srtm.csi.cgiar.org/) [21].

Yixing (31°07´N–31°37′N, 119°31′E–120°03′E) locates in southern Jiangsu, which is south of the lower Yangtze River Basin and west of Tai Lake (Fig 1). The variation of the latitude (0–611 m a.s.l.) leads to uneven population distribution in the county. Relatively few people settle in the southern mountainous areas (the north piedmont of the Tianmu Mountain); most the people inhabit the northern plains areas. The total area of Yixing is 2038.7 km^2^, while the total water area in Yixing reaches 532.6 km^2^. Several lakes are scattered in Yixing, including Tai Lake, Ge Lake, Xijiu Lake, Tuanjiu Lake, Dongjiu Lake, and more. There are 215 waterways, including the Liyi River, Mengjin River, Wuyi Canal, Nanxi River, Beixi River, Wuxi River, Taoxi River, Li River, Hengtang River, Caoqiao River, and others. Their total length is up to 1,058 km. Influenced by Asian Summer Monsoon, the climate in Yixing is a sub-tropical one: the mean annual rainy days are 136.6 in number (mainly rains in spring and summer); mean annual precipitation is 1,177 mm; mean annual temperature is 15.7°C; and mean annual summer temperature is 28.3°C.

Yixing County was populated during the Ming and Qing dynasties. Population size increased from 165,223 to 287,912 in AD1434–1882 [the Old Local Chronicles of Yixing County (*Yixing Jiuzhi*), Compendium of China’s local Chronicles (*Zhongguo Difangzhi Jicheng*), and County Annals on Yixing and Jinxi County (*Yixing Jinxi Xianzhi*)].

## Data and methods

### Archaeological investigation

**Fig 2:**
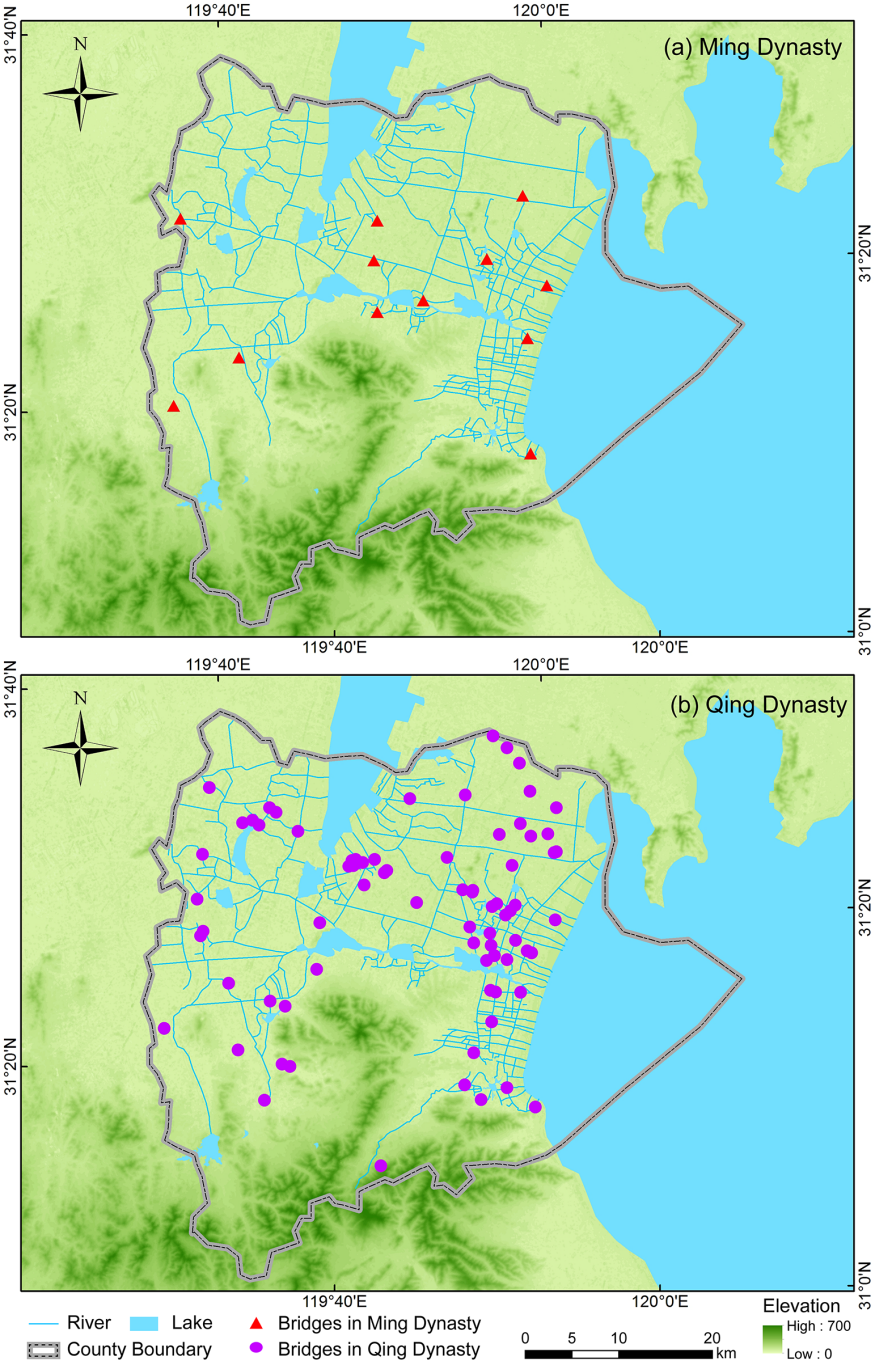
Geographic distribution of the Ming and Qing bridges in Yixing. The maps were generated in ArcGIS version 10.1 (www.esri.com/software/arcgis).

We surveyed all of the ancient bridges in Yixing in our field investigations during 2015–2016. There are 89 ancient bridges in total. Twelve of them were built during the Ming dynasty (AD1368–1644) and 77 of them were built during the Qing dynasty (AD1644–1911) (Fig 2). General information about the two dynasties is presented in Table A in S1 File. The construction period of ancient bridges was determined according to their traditional typologies, inscriptions, and associated historical documents. We crosschecked our ancient bridge survey records with historical documents such as local chronicles and county annals, and found that most of the important bridges in ancient times (e.g., Jingtang bridge, Bulong bridge, etc.), which are mentioned in historical documents, are also found in our records. We obtained accurate locational information for each bridge using global positioning system (GPS) receivers during our field investigations. Other related bridge information was also recorded, including their length, width, height, construction material, and number of apertures. Our complete survey records are available in Table B in S1 File.

### ArcGIS spatial analysis

**Fig 3:**
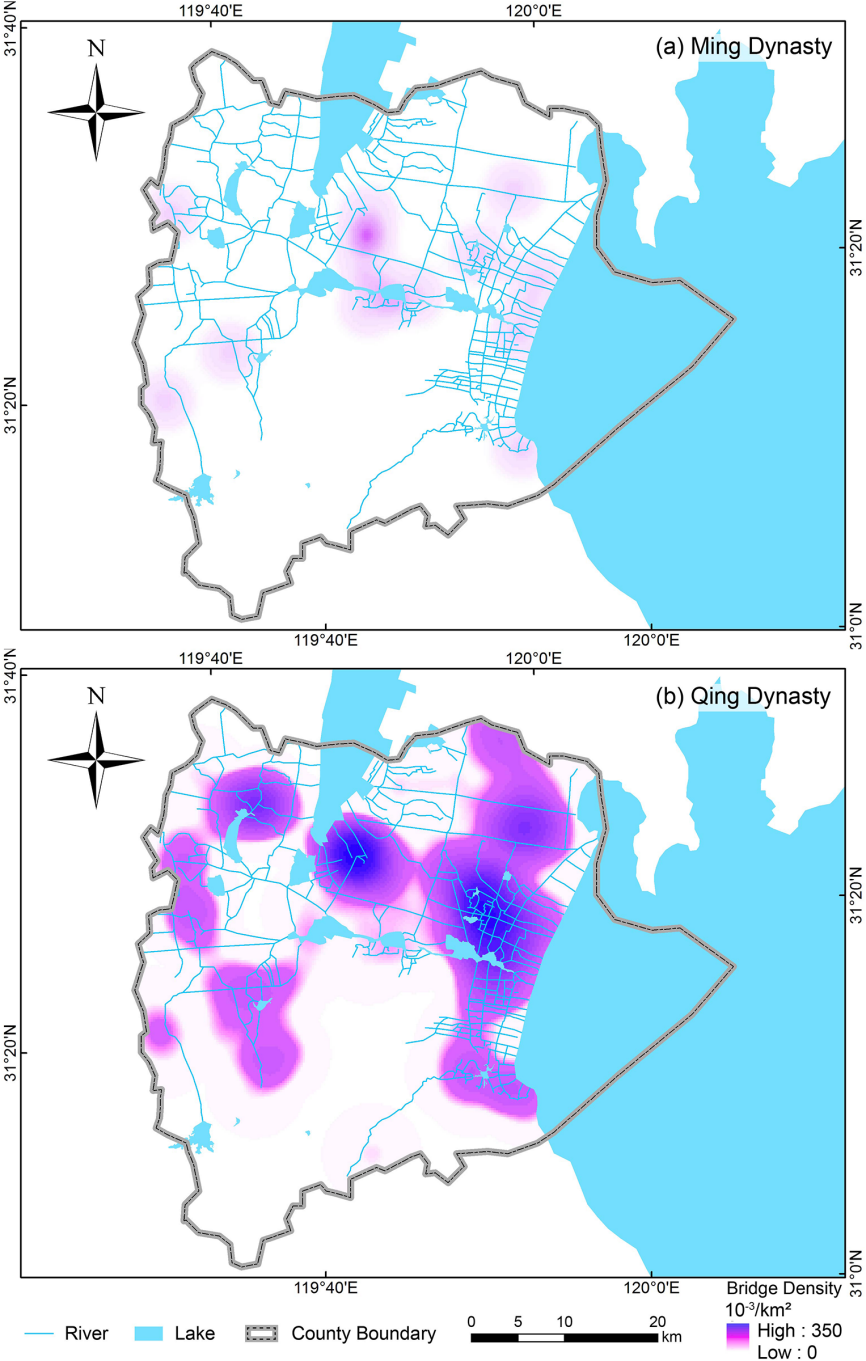
Density maps of the Ming and Qing bridges in Yixing. The maps were generated in ArcGIS version 10.1 (www.esri.com/software/arcgis).

ArcGIS was employed for the spatial analysis of ancient bridges. The GPS information of every bridge was input into ArcGIS. The density distribution (Di) was calculated by the function “Spatial Analyst Tools—Density—Kernel Density” in ArcGIS version 10.1; the same function was employed to make density maps. Under this method, a cluster (i.e., geographic concentration of bridges) is determined by the number of bridges which fall within a neighborhood around each cell. In this study, the radius parameter of neighborhood was optimized as 5 km in calculating the geographic concentration of ancient bridges (Fig 3).

### The estimation of population density and distribution using ancient bridges

The construction of bridges was determined by the volume of traffic. In addition, bridges were often the hub of land and water transportation. They were also checkpoints for tax collection and local administration [22]. For example, a checkpoint was set up around the Nan Bridge in Fengxian in Shanghai for tax collection [23]. Once the bridges were built, people also tended to settle somewhere near the bridges because they provided good accessibility [24]. Subject to the above circumstances, the distribution and structure of bridges can be useful indicators in inferring population density and distribution in a region. Therefore, we based on the distribution and structure of ancient bridges in our survey records to project the population growth dynamics (in terms of population density and distribution) in Yixing during in the Ming dynasty and Qing dynasty, respectively.

**Fig 4:**
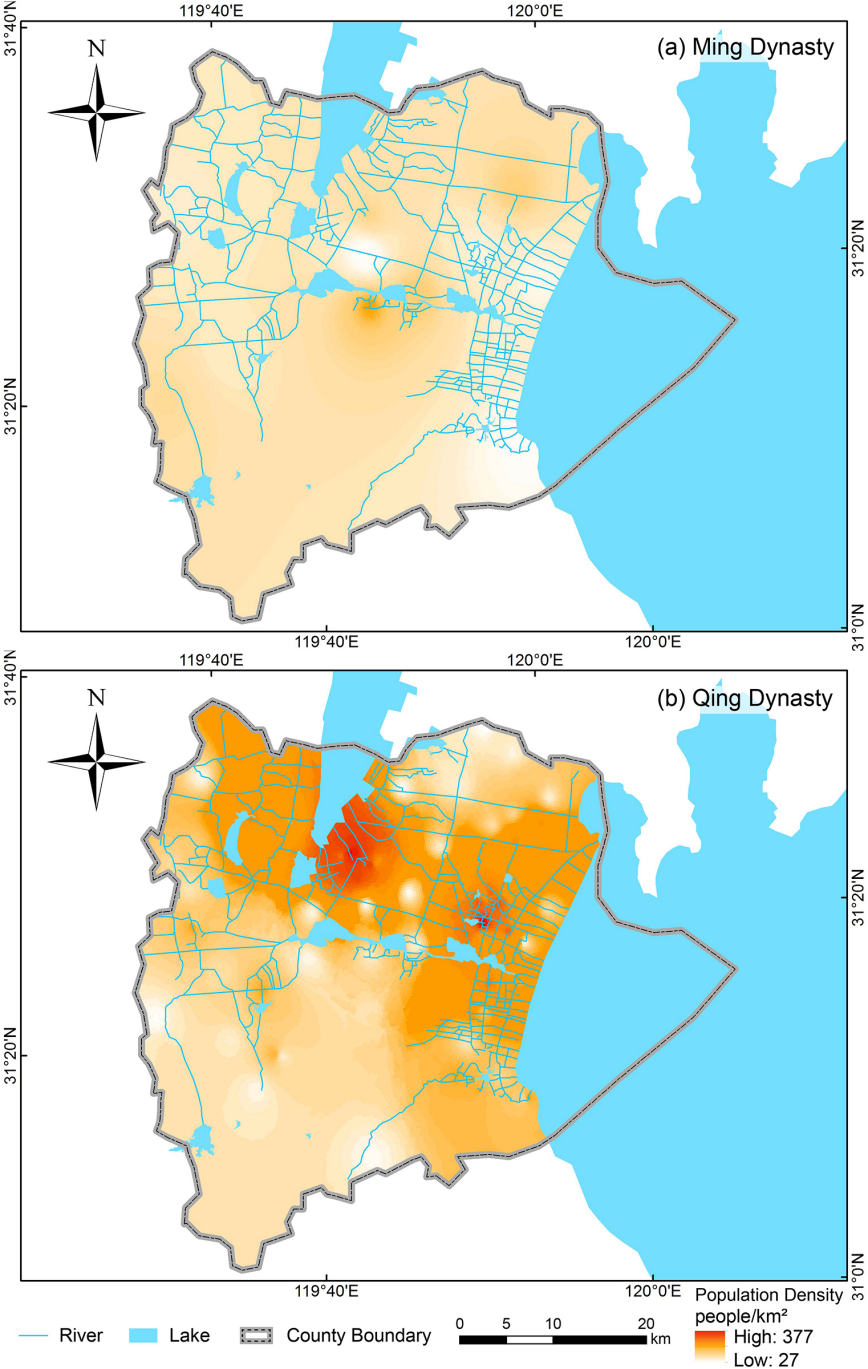
Projection of population density and distribution in Yixing in Ming and Qing dynasties. The maps were generated in ArcGIS version 10.1 (www.esri.com/software/arcgis).

First, the average population as reflected by ancient bridges ($\bar{\text{P}}$) was calculated by dividing the total population size by the total number of ancient bridges. Second, the first coefficient (X_i_) was calculated by dividing the width of each bridge (W_i_) by the average width of bridges ($\bar{\text{W}}$). Third, the distribution density of each bridge (D_i_) was extracted from ArcGIS. Then, the second coefficient (Y_i_) was calculated by dividing the distribution density of each bridge (Di) by the average distribution density of bridges ($\bar{\text{D}}$). Fourth, the population associated with each bridge (P_i_) was obtained by multiplying ($\bar{\text{P}}$) with the coefficients (X_i_ and Y_i_). Finally, all of the values of (P_i_) were input into ArcGIS according to their location, and the density distribution of population was generated by “Kriging interpolation” (Fig 4).

We also compared the areal density of ancient bridges with the areal density of waterways in the Ming and Qing dynasties, respectively, to check the extent to which the number of ancient bridges is determined by the number of waterways. Only a very weak correlation was found (Figure A in S1 File). This further confirms that the presence of bridges is mainly determined by population size rather than by the number of waterways that need to be crossed. This may further justify the above method in projecting population density and distribution using ancient bridges.

## Results

### Geographic distribution of Ming and Qing bridges in Yixing

There are 89 ancient bridges in Yixing. The number of ancient bridges built in the Qing dynasty is 77 (Fig 2-b), which is 6.42 times more than were built in the Ming dynasty (12, Fig 2-a). Those ancient bridges were mainly found in the northern plains regions in Yixing (Fig 2). The 12 Ming bridges are scattered in the northern region of Yixing where rivers and lakes crisscross. The geographic distribution of bridges stretched out over time. Although most ancient bridges were located in the northern region of Yixing, there were a few bridges in the southern mountainous region. The Qingliang Bridge locates in Hufu, the southernmost mountain region in Yixing. At the same time, the geographic distribution of ancient bridges was also slightly extended northward. Huanggantaiping Bridge locates in Heqiao, which is the northernmost plains region in Yixing. As illustrated in Figs 2 and 3, several of the ancient bridges cluster in the lakefront areas (less than 5 km from the lakeside). Furthermore, the elevation of nearly all of the ancient bridges is below 10 m a.s.l.

### Structural differences of the Ming and Qing bridges in Yixing

The ancient bridges were constructed with stone, including marble and bluestone. Xiafang Bridge, which locates in Fangqiao, displays the greatest number of arches (four arches). There are 78 single-arch stone bridges (11 of them were built in the Ming dynasty and 67 of them were built in the Qing dynasty) and 10 three-arch stone bridges (one of them was built in the Ming dynasty and nine of them were built in the Qing dynasty). As shown in Table 1, the average length of the Ming bridges is 21.44 m, which is 2.85 m longer than the Qing bridges (18.59 m). The longest bridge is Jingtang Bridge, which is 52 m in length and was built in the Ming dynasty. The average width of the Ming bridges is 2.86 m, which is a bit wider than the Qing bridges (2.77 m). The shortest bridge is Gezhuangshuangqiao Bridge, which is 4.7 m length and was built during the Qing dynasty. The widest (Zhangze Bridge) and the narrowest bridges (Wan’an Bridge) were also built in the Qing dynasty, with widths of 4.7 m and 0.46 m, respectively.

**Table 1:** Structural difference of Ming and Qing bridges in Yixing.

Dynasty	Ming	Qing
Number	12	77
Average length (m)	21.44	18.59
Shortest (m)	10.20	4.70
Longest (m)	52.00	45.95
Length<10 m	0	6
Length [10 m, 20 m]	8	45
Length>20 m	4	26
Average width (m)	2.86	2.77
Narrowest (m)	1.68	0.46
Widest (m)	3.95	4.70
Width<2.5 m	3	25
Width≥2.5 m	8	50

### Projection of the density and distribution of population in Yixing in the Ming and Qing dynasties

On the basis of the density distribution and width of the ancient bridges, we estimated the population density in Yixing during the Ming and Qing dynasties. In general, population was primarily concentrated in the northern plains regions in Yixing. Yet, population distribution in the Ming dynasty is different from that in the Qing dynasty. During the Ming dynasty, the most densely populated region located in the south bank of the junction between Xijiu Lake and Tuanjiu Lake (112 people/km^2^), while the second most densely populated region probably located in Fangqiao (100 people/km^2^). The most densely populated region shifted northeastward to Xinzhuang (377 people/km^2^) during the Qing dynasty. The second most densely populated region locates in Gaocheng (355 people/km^2^) on the east bank of Ge Lake. Synchronously, some people migrated southeastward and settled in Dingshu in the southern part of the west bank of Tai Lake (192 people/km^2^).

## Discussion

### Factors determining the number of ancient bridges

A bridge was one of the major transport facilities in Jiangnan, a region characterized by interwoven river networks. It is functionally similar to a ship, which helps people travel to the other side of rivers and lakes. The increasing number of bridges between the Ming and Qing dynasties (6.41 times increase) reveals the growth of traffic in Yixing. Furthermore, the increase was probably ascribed to population expansion in Yixing. Population size in Yixing increased from 165,223 (AD1434) to 287,912 (AD1882) between the Ming and Qing dynasties. In AD1726, Yixing was split into two counties, namely Yixing and Jingxi, probably owing to the management challenges caused by population growth.

LIA was a period of abnormal cooling in the Late Holocene on the centennial time scale, and corresponds approximately to the Ming and Qing dynasties in Chinese history. Annual mean temperature in LIA was 1–2°C lower than that in the modern era [25]. People migrated southward in huge numbers from northern China because of the cold climate. The mean population center in China moved to the south, and the North-South population ratio decreased during the time, which is confirmed by statistical analysis [26–27]. Together with the social development that occurred in Jiangnan when Nanjing was chosen as the capital of China in the Ming dynasty, this led to the growth of traffic in the Jiangnan region. People had to build more bridges for waterway transportation to augment boats. Hence, twelve bridges were built in Yixing during the Ming dynasty. In the early stage of this migration (Ming dynasty), human settlement clustered at some locations that were proximate to a waterway transportation network. However, the population growth in Yixing was halted in the 17^th^ century, the coldest century over the past two millennia. In the lower Yangtze River Basin, annual mean temperature was 1°C lower than that in the 20^th^ century [28]. This cooling event triggered various mortality crises such as war, famine, and epidemics, resulting in population decline in Jiangnan. According to the County Annals on Yixing and Jingxi (*Yixing Xianzhi* and *Jingxi Xianzhi*), the population in Yixing was only around 148,660 in AD1645, which is even less than the population in AD1434 (165,223).

The annual mean temperature of Jiangnan in the 18^th^ century was 1°C higher than that in the 17^th^ century [29]. The warmer climate boosted agricultural harvests, which facilitated rapid population growth. With the growth of population in Yixing in the 18^th^ century, people had to expand their living areas. At the same time, the increase in population size and the limited land area in Yixing resulted in surplus farm laborers [30–31]. People were pushed to engage in industry and commerce to earn money for food. Hence, primitive industry and commerce emerged in Yixing. The growth of traffic in Yixing was reinforced by the population increase and the development of industry and commerce. This pushed people to build additional bridges for waterway transport. To meet the traffic demand and accommodate social development, 77 bridges were built in Yixing during the Qing dynasty.

Indeed, the population growth in Yixing and Jiangnan was synchronous. The population in Jiangnan region was about 8.7 million in the early Ming dynasty (AD1393). It reached 20 million in the late Ming period (AD1620). This was caused by the huge number of migrants from northern China. Population reduced to the low of 15.25 million in AD1644 because of food shortages, frequent wars and massive death tolls. Yet, population size bounced back to 20 million in AD1680. In conjunction with the mild climate and social stability, the population in the Jiangnan region increased to 36 million in the late Qing period (AD1850) [32–33]. The socio-economic development in the Qing dynasty was reflected in population increase, as the growth of population promoted early simple industry and commerce in the Jiangnan region [34–36]. The increasing population was not only associated with more frequent flows of people, but also with more frequent flows of goods. Therefore, more bridges were also built in Jiangnan in the Qing dynasty.

### Factors determining the structure of ancient bridges

Nearly all of the ancient bridges in Yixing were constructed with stone, particularly marble and bluestone. These materials were probably obtained from the Tianmu Mountains in the south of Yixing. The accessibility of construction materials reduced the cost of bridge building, which facilitated the construction of bridges in Yixing. On the other hand, the construction of bridges was often accompanied by stone mining and transportation. The increasing number of bridges also implied the growth of the labor force and socio-economic well-being between the Ming and Qing dynasties.

Locations of bridges were determined by traffic demand. Using the same rubric, the structure of ancient bridges was probably linked with the socio-economic situation during the time of their construction. Jingtang Bridge, the longest bridge built in the Ming dynasty, was an important transportation and communication route during the Ming and Qing dynasties. People had to cross this bridge to go to Liyang. This road extended westward to Nanjing, connecting local and central administration together. The construction of other bridges probably met the growing traffic demand during the Ming dynasty [37–38]. On the other hand, although the number of bridges increased in Yixing between the Ming and Qing dynasties, more short (less than 10 m) and narrow (less than 2.5 m) bridges were built during the Qing dynasty (Table 1). The average length of bridges decreased from 21.44 m to 18.59 m and the average width decreased from 2.86 m to 2.77 m. The narrowest bridge built in the Ming dynasty was 1.68 m in width, while one constructed in the Qing dynasty was only 0.46 m in width, which is just wide enough for a person to pass across. In the Qing dynasty, increasing numbers of people settled in Yixing, but the population was dispersed across different parts of the region. Many small settlements were formed. For those settlements with limited numbers of people, small (i.e., short and narrow) bridges would be sufficient to cater to the associated transportation needs. The trend of building smaller (shorter and narrower) bridges helps explain why the increase in the number of bridges (6.41 times) is much larger than the increase of population size (1.74 times) in Yixing between the Ming and Qing dynasties (cf. Factors determining the number of ancient bridges), as smaller bridges were required to cater for the growing number of small settlements, as well as rapid population growth/dispersal in the Qing dynasty.

### Ancient bridges and the density and distribution of population

According to our projection, during the Ming dynasty, the most densely populated region (112 people/km^2^) located in the south bank of the junction between Xijiu Lake and Tuanjiu Lake, which was also the location of local government, as well as a local political and economic center during the time. Wanshi (100 people/km^2^) and Gaocheng (95 people/km^2^) were the second most densely populated regions. Their formation is probably attributable to the growth of clan population there. Regarding the clans’ decision to migrate, when the push factors became unbearable, all of the people in the same clan would migrate together. This phenomenon contributed to economic development, population growth, social stability, and facilities construction in the destinations in the lower Yangtze River Basin. The Clans of Wang, Zhou, Jiang, Wu, Chen, and Du contributed to high population density in Gaocheng [Annals of the Gaocheng Town (*Gaocheng Zhenzhi*)]. In addition, clans rather than government were responsible for building schools and providing public facilities (including bridges) at the local level, which sustained the local economy [39–40]. In the Ming dynasty, Shitang and Bulong Bridges were built by the Clans of Yu and Ren, respectively. In the Qing dynasty, Nancaotang Bridge was re-built by the Clan of Chu [County Annals of Jingxi (*Jingxi Xianzhi*)]. According to the inscriptions of Jingtang Bridge, which is the longest ancient bridge in Yixing, the bridge was built and financed by the Clan of Qian in AD1551. The bridge was also revamped by the same clan in AD1686.

In the Qing dynasty, the most densely populated region shifted northeastward to Xinzhuang (377 people/km^2^). The second most densely populated region located on the southeast bank of Ge Lake (355 people/km^2^). The population of different clans increased rapidly in the Qing dynasty. Similar to the example of Gaocheng mentioned in the previous paragraph, the rapid population growth of clans transformed those locations with the cluster of clans to densely populated regions.

Simultaneously, some of people migrated southeastward to Dingshu in the southern part of the west bank of Tai Lake (192 people/km^2^) because of the blossoming of Yixing Teapot (Zisha Teapot) handicrafts [41]. Yixing Teapots and other art wares have been renowned at home and abroad since the Qing dynasty. The growth of the Yixing Teapot handicraft industry during the Qing dynasty significantly improved the socio-economic well-being of people in Dingshu during the period, which attracted more people to settle there for the making of Yixing Teapots.

## Conclusion

In Yixing, 77 bridges were built during the Qing dynasty, which is 6.41 times more than the number built during the Ming dynasty (12). The increasing number of bridges was attributable to escalating traffic demands, which was stimulated by the growth of population over time. The location and the structure of ancient bridges were determined by the volume of traffic. In the Ming dynasty, bridges were built on pivotal routes; in the Qing dynasty, owing to the dispersal of population, bridges scattered across various places in Yixing. The decreasing average length and width of bridges between the Ming and Qing dynasties was probably due to the dispersal of population and the associated formation of small settlements in the latter period. Hence, smaller (i.e., short and narrow) bridges would be sufficient to cater to the needs of the more limited flow of people. The emergence of settlement hotspots in Yixing was accompanied by the massive migration of clans from northern China during the LIA. The growth of clans’ population drove the socio-economic development and the provision of infrastructure at the local level. Increasing numbers of bridges were built around the settlements of large clans. In addition, the growth of population density in Yixing was also attributable to the blossoming of a commodity economy, which was represented by the making of Yixing Teapots. In this study, the archaeological records of ancient bridges were employed to recover the density and distribution of population in Yixing over an extended period. Our approach is innovative and robust, and could be employed to recover the long-term historical population growth dynamics in other parts of China.

## Supporting information

S1 File Supplementary figures and tables. (DOCX)
