# Supplementary material for: Relationship between ancient bridges and population dynamics in the lower Yangtze River Basin, China
Source: PLoS One. 2017 Aug 9;12(8):e0182560. doi: 10.1371/journal.pone.0182560 (PMC5549911; doi:10.1371/journal.pone.0182560)
Supplement: S1 File — (DOCX) [file pone.0182560.s001.docx]

**S1 File. Supplementary figures and tables.**

This file contains:

**Fig A. Correlation between drainage density and ancient bridge density in Yixing.** (a) Ming dynasty; (b) Qing dynasty.

**Table A. General information of the Ming and Qing dynasties.**

**Table B. Survey records of ancient bridges in Yixing that were built in the Ming and Qing dynasties.**


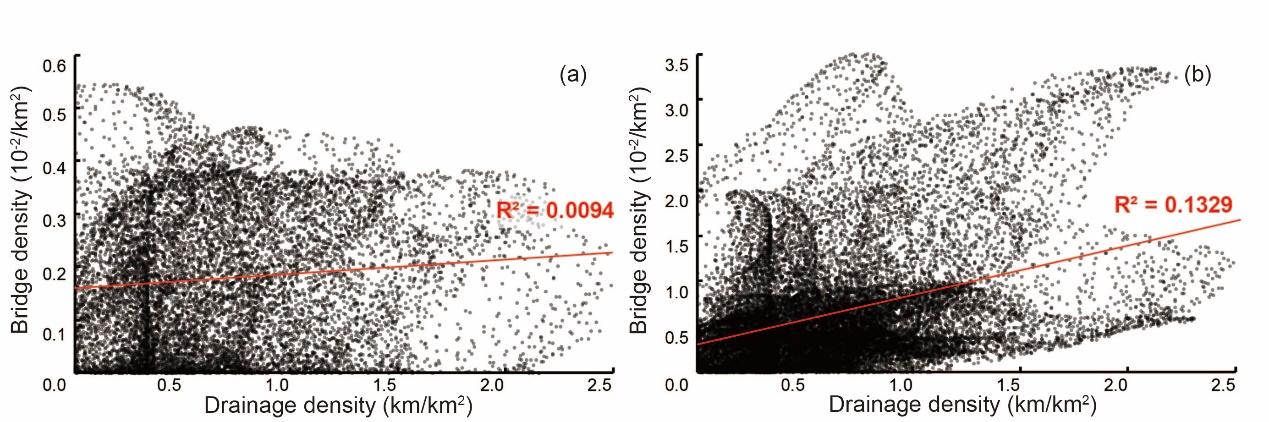


**Fig A. Correlation between drainage density and ancient bridge density in Yixing.** (a) Ming dynasty; (b) Qing dynasty.

**Table A. General information of the Ming and Qing dynasties.**

| **Dynasty** | **Period** | **Capital** | **Population size^1^ (million)** |
| --- | --- | --- | --- |
| Ming | AD1368–1644 | Nanjing (1368－1421; 1424－1441)  Beijing (1421－1424; 1441－1644) | 60 (AD1370)  160 (AD1620) |
| Qing | AD1644–1911 | Beijing | 90 (AD1650)  100 (AD1680)  200 (AD1740)  374 (AD1810)  450 (AD1850)  370 (AD1870)  443 (AD1900) |

1 Population data are obtained from Jiang ([1993](#_ENREF_1)).

**Table B. Survey records of ancient bridges in Yixing that were built in the Ming and Qing dynasties.**

| **Name** | **Longitude** | **Latitude** | **Length** | **Width** | **Material** | **Age** |
| --- | --- | --- | --- | --- | --- | --- |
| Bulong bridge | 119.5465 | 31.3143 | 19 | 3.3 | Bluestone | 1573 |
| Dacheng Taiping bridge | 119.8971 | 31.3914 | 10.1 | 2.55 | Bluestone | Ming |
| Dapu bridge | 119.9234 | 31.3145 | 27.2 | 2.7 | Bluestone, Marble, Yangshan stone | Ming |
| Dongcang bridge | 119.8235 | 31.3656 | 30 | 3.6 | Bluestone, Marble | Ming |
| Fufeng bridge | 119.9466 | 31.4413 | 19.05 | 3.55 | Bluestone, Marble | Ming |
| Jiaodu bridge | 119.9535 | 31.358 | 14.7 | 2.4 | Bluestone, Marble, Yangshan stone | Ming |
| Jingtang bridge | 119.6228 | 31.3461 | 52 | 4.5 | Bluestone, Marble | Ming |
| Shengping bridge | 119.9039 | 31.2125 | 14 | 2.1 | Bluestone, Marble | Ming |
| Xifang bridge | 119.7805 | 31.4093 | 10.2 | 1.68 | Bluestone, Marble, Yangshan stone | Ming |
| Xin bridge | 119.7739 | 31.3631 | 31.3 | 3.95 | Bluestone, Marble | Ming |
| Yangxi bridge | 119.5894 | 31.4783 | 18.2 | 2.7 | Bluestone, Marble | Ming |
| Yongan bridge | 119.792 | 31.4437 | 11.5 | 2.9 | Bluestone, Marble | Ming |
| Caojia bridge | 119.9159 | 31.3868 | 36.4 | 2.9 | Bluestone | Qing |
| Changan bridge | 119.8561 | 31.2706 | 13.6 | 2.75 | Bluestone, Marble | 1882 |
| Changgong bridge | 119.5425 | 31.3440 | 9.8 | 1.9 | Bluestone | Qing |
| Dayu bridge | 119.6796 | 31.5077 | 23.28 | 3.5 | Bluestone, Marble | Qing |
| Dongtang bridge | 119.8023 | 31.4461 | 25.6 | 3.05 | Marble | Qing |
| Fenghuang bridge | 119.8544 | 31.2281 | 28.8 | 3.8 | Bluestone, Marble, Yangshan stone | Qing |
| Fu bridge | 119.8861 | 31.4126 | 18.6 | 0.38 | Bluestone | Qing |
| Futian bridge | 119.5978 | 31.4197 | 34.6 | 3.95 | Bluestone, Marble | Qing |
| Gezhuang big bridges | 119.6939 | 31.5213 | 4.7 | 2.22 | Bluestone | Qing |
| Gezhuang little bridge | 119.6939 | 31.5213 | 22.1 | 3.16 | Bluestone, Marble, Yangshan stone | Qing |
| Gujia wanshou bridge | 119.9254 | 31.4590 | 15 | 1.79 | Bluestone, Marble | Qing |
| Guanlin bridge | 119.7186 | 31.4957 | 25.4 | 3.75 | Bluestone | Qing |
| Guijing bridge | 119.6713 | 31.3433 | 17.18 | 3.5 | Bluestone, Marble | Qing |
| Houting bridge | 119.8669 | 31.4475 | 12 | 2.75 | Bluestone, Marble | 1899 |
| Huaxi bridge | 119.8403 | 31.2440 | 34.94 | 4 | Bluestone, Marble | Qing |
| Huanggan bridg | 119.7763 | 31.4373 | 11.95 | 3.15 | Bluestone, Marble | 1894 |
| Huanggan taiping bridge | 119.6356 | 31.5491 | 14.4 | 2.9 | Bluestone, Marble | Qing |
| Huisheng bridge | 119.8834 | 31.2343 | 12.75 | 2.5 | Bluestone, Marble | Qing |
| Jiangdian bridge | 119.6016 | 31.4525 | 36.26 | 2.16 | Bluestone, Marble | Qing |
| Jiangjia bridge | 119.6997 | 31.5162 | 15.8 | 3.2 | Bluestone, Marble, Yangshan stone | Qing |
| Jinlong bridge | 119.6317 | 31.2639 | 6 | 2.5 | Bluestone | Qing |
| Jinpao bridge | 119.6645 | 31.2894 | 12.3 | 1.8 | Marble | Qing |
| Jubao bridge | 119.8963 | 31.3528 | 13.15 | 1.68 | Bluestone, Marble, Yangshan stone | Qing |
| Masidu bridge | 119.8403 | 31.5058 | 13 | 2.6 | Bluestone | Qing |
| Matang bridge | 119.9324 | 31.4294 | 21.4 | 3.3 | Bluestone, Marble, Yangshan stone | Qing |
| Meiqian yongxing bridge | 119.9216 | 31.3898 | 17.6 | 3.08 | Bluestone, Marble | 1887 |
| Nancaotang bridge | 119.8767 | 31.4164 | 22.2 | 3.7 | Bluestone, Marble | Qing |
| Qinglong bridge | 119.9808 | 31.4339 | 16.1 | 2.75 | Marble | Qing |
| Qingliang bridge | 119.7386 | 31.1867 | 11 | 2.8 | Yellowstone | Qing |
| Shangtang bridge | 119.8767 | 31.3822 | 29.44 | 3.15 | Bluestone | Qing |
| Shidang bridge | 119.8806 | 31.2949 | 19.9 | 3.33 | Bluestone, Marble | Qing |
| Shitang bridge | 119.8856 | 31.3228 | 24.8 | 3.58 | Bluestone | Qing |
| Shuangwei bridge | 119.6568 | 31.3504 | 31 | 3.8 | Bluestone, Marble | Qing |
| Tangjiao yongan bridge | 119.8949 | 31.3624 | 23.2 | 1.7 | Bluestone, Marble | Qing |
| Tongzi bridge | 119.6739 | 31.5130 | 14.5 | 2.96 | Bluestone | Qing |
| Wanan bridge | 119.9352 | 31.3491 | 25 | 0.46 | Marble | Qing |
| Wanxing bridge | 119.9309 | 31.3516 | 15.9 | 1.9 | Bluestone, Marble | Qing |
| Wanyuan bridge | 119.9755 | 31.4513 | 20.5 | 3.5 | Marble | Qing |
| Wangpo bridge | 119.8778 | 31.3677 | 28.45 | 3.4 | Bluestone, Marble, Yangshan stone | Qing |
| Weidu bridge | 119.9085 | 31.3471 | 20 | 1.8 | Bluestone, Marble, Yangshan stone | Qing |
| Wuze bridge | 119.6015 | 31.4233 | 24.45 | 2.4 | Bluestone, Marble | Qing |
| Wuchang bridge | 119.9781 | 31.4337 | 9.85 | 2.9 | Bluestone, Marble | Qing |
| Xiyan bridge | 119.9492 | 31.4650 | 13.7 | 1.37 | Marble | Qing |
| Xiatian bridge | 119.7993 | 31.4447 | 11.7 | 2.4 | Marble | Qing |
| Xiafang bridge | 119.9576 | 31.4521 | 20 | 1.75 | Marble | Qing |
| Xianbu bridge | 119.6144 | 31.3126 | 22.8 | 3.1 | Bluestone, Marble | Qing |
| Xianlong bridge | 119.9210 | 31.3627 | 15.85 | 2.7 | Bluestone, Marble | Qing |
| Xiangda bridge | 119.8267 | 31.4128 | 12.5 | 2.4 | Bluestone | Qing |
| Xindu yongan bridge | 119.7922 | 31.4580 | 24 | 2.75 | Marble | Qing |
| Xinglong bridge | 119.7734 | 31.4576 | 15 | 3.2 | Bluestone, Marble | Qing |
| Xiuning bridge | 119.7791 | 31.4572 | 12.58 | 2.9 | Bluestone, Marble | Qing |
| Xujia yongxing bridge | 119.7232 | 31.4114 | 22.3 | 3 | Bluestone, Marble | 1887 |
| Xuanzhuang bridge | 119.9603 | 31.5184 | 15.3 | 1.35 | Marble | Qing |
| Xueyin bridge | 119.9162 | 31.3161 | 13.24 | 3.38 | Bluestone, Marble | Qing |
| Yanzhuang peide bridge | 119.9656 | 31.4918 | 23.6 | 2.84 | Bluestone, Marble | Qing |
| Yangtang bridge | 119.9092 | 31.3984 | 12.1 | 2.65 | Bluestone | Qing |
| Yangxi bridge | 119.9661 | 31.3740 | 12.2 | 2.58 | Marble | Qing |
| Yaoxi bridge | 119.6158 | 31.4912 | 28 | 3.95 | Bluestone, Marble | Qing |
| Yongfeng bridge | 119.7688 | 31.4561 | 14.35 | 2.8 | Bluestone, Marble | Qing |
| Yongji bridge | 119.8963 | 31.3734 | 17.6 | 3.5 | Bluestone, Marble | Qing |
| Yongji bridge | 119.7640 | 31.4563 | 18.93 | 2.45 | Bluestone, Marble | Qing |
| Yongnian bridge | 119.7685 | 31.4609 | 13.85 | 2.8 | Bluestone, Marble | Qing |
| Yongning bridge | 119.8872 | 31.4141 | 16.2 | 2.5 | Bluestone, Marble, Yangshan stone | 1738 |
| Yongsi bridge | 119.7109 | 31.3708 | 15.9 | 3 | Bluestone | Qing |
| Yongxing bridge | 119.7726 | 31.4613 | 10.28 | 2.8 | Marble | Qing |
| Yucheng bridge | 119.9385 | 31.5470 | 18.55 | 1.12 | Bluestone, Marble, Yangshan stone | Qing |
| Yudai bridge | 119.6569 | 31.2928 | 16.1 | 3.8 | Bluestone, Marble | Qing |
| Yulan bridge | 119.9085 | 31.2123 | 18 | 3.9 | Bluestone | Qing |
| Yunji bridge | 119.8982 | 31.4995 | 19.4 | 3.6 | Bluestone, Marble | Qing |
| Zhangze bridge | 119.8903 | 31.3206 | 45.95 | 4.7 | Bluestone, Marble | Qing |
| Zhenlong bridge | 119.6633 | 31.5125 | 20.63 | 3.1 | Bluestone | Qing |
| Zhouluo bridge | 119.9504 | 31.5341 | 15.5 | 1.6 | Bluestone, Marble | Qing |
| Zhuheng bridge | 119.9277 | 31.3938 | 16.84 | 1.7 | Bluestone, Marble, Yangshan stone | Qing |
| Zhutenghua bridge | 119.9894 | 31.4729 | 13 | 1.5 | Marble | Qing |
| Zhuan bridge | 119.6176 | 31.3730 | 15.2 | 3.3 | Bluestone | Qing |
| Zhuangqian bridge | 119.9038 | 31.3966 | 8.85 | 1.8 | Bluestone, Marble | Qing |
| Zunxian bridge | 119.8873 | 31.3498 | 8.8 | 2.6 | Bluestone, Marble | Qing |

**Reference:**

Jiang T. (1993) *Zhongguo Jindai Renkou Shi [Recent Population History in China],* Hangzhou: Hangzhou University Press.
